# Study on the Combined Effect of Municipal Solid Waste Incineration Bottom Ash and Waste Shingle in Hot Mix Asphalt

**DOI:** 10.3390/ma17010046

**Published:** 2023-12-21

**Authors:** Kyungwon Park, Behnam Golestani, Boo Hyun Nam, Juan Hou, Jongwan Eun

**Affiliations:** 1 Department of Civil Engineering, College of Engineering, Kyung Hee University, Yongin 17104, Republic of Korea; kwpark@khu.ac.kr; 2 Intertek-PSI, 1748 33rd St., Orlando, FL 32839, USA; ben.gol@intertek.com; 3 School of Mechanics and Engineering Science, Shanghai University, Shanghai 200444, China; juanhou@staff.shu.edu.cn; 4 Department of Civil & Environmental Engineering, University of Nebraska-Lincoln, Lincoln, NE 68588, USA; jeun2@unl.edu

**Keywords:** hot mix asphalt, municipal solid waste incineration (MSWI) bottom ash, recycled asphalt shingle

## Abstract

This study investigated the positive effect of the combined use of recycled asphalt shingles (RASs) and municipal solid waste incineration (MSWI) bottom ash (B.A.) in asphalt concrete, which contributes to enhanced sustainability in pavement engineering. In addition, unlike traditional approaches that employ individual recycling material in hot mix asphalt (HMA), the combined use of the two waste materials maximizes the mechanical performance of the asphalt mixture. The addition of RAS (with 30–40% aged binder) as an additive generally enhances the strength/stiffness of the asphalt mixture. The high porosity/absorption of MSWI BA results in an additional amount of liquid asphalt binder in the mixture. As an admixture, RAS could supply the additional asphalt binder in the mixture when MSWI BA is used as an aggregate replacement. This research was conducted in two phases: (1) to examine the effect of MSWI BA alone and its optimal asphalt content (OAC), and (2) to assess the combined effect of B.A. and RAS in HMA. Multiple laboratory testing methods were employed for the mechanical performance investigation, including the Marshall stability test, rutting test, and indirect tensile test. The testing results show that the 20% B.A. replacement exhibits the best performance and that it requires an additional asphalt binder of 1.1%. For the combined use of MSWI BA and RAS, 5% RAS shows the best mechanical performance. All mixtures that contain the B.A. and RAS show greater strength than the control specimen (regular HMA).

## 1. Introduction

The increase in the global population has resulted in a significant rise in the amount of waste generated, leading to limited landfill space and increasing the cost of waste disposal. To address this issue, considerable efforts have been made to adopt sustainable material solutions, such as recycling waste products in various engineering applications [1]. One such area of interest is to burn municipal solid waste for the energy generation and management of incineration ash. Several countries have been effectively managing the MSWI (municipal solid waste incineration) bottom ashes through various implementation programs and regulations [2,3,4,5,6].

**Figure 1:**
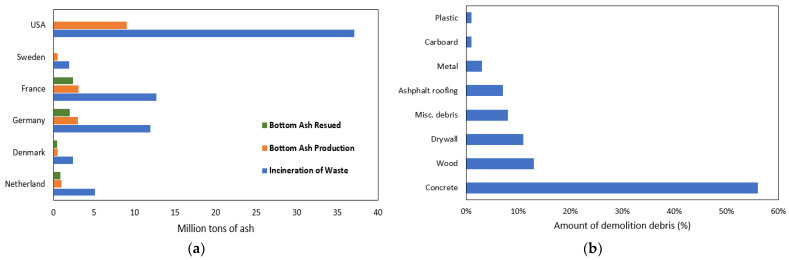
(**a**) Comparison of the management of MSWI BA (Cho et al. [10]) and (**b**) the component of construction and demolition debris (Nam et al. [16]).

In compliance with environmental standards set forth by national regulations, many European countries have adopted the use of MSWI bottom ash (B.A.) as a sustainable material for transportation applications [3,4]. Figure 1a compares MSWI BA generation and its recycling rate for several countries. Interestingly, despite the larger production of municipal solid waste (MSW) in the U.S. compared to any other country, the recycling rate in the U.S. remains surprisingly low. Since 1980, the total amount of MSW generated in the U.S. has risen by 65%, resulting in an annual volume of 250 million tons, of which 53.6% is disposed of in landfills, 34.7% is recycled, and 11.7% is used for energy generation through incineration [7]. In the U.S., eighty-six MSW incineration plants were being operated over twenty-four states [8], with New York, New Jersey, Connecticut, Pennsylvania, and Virginia as the primary users of MSWI plants [6]. Those incineration facilities’ usual by-products include MSWI fly ash (F.A.) and bottom ash (B.A.). As a common practice of MSWI management, the fly ash, which is treated as hazardous waste, is combined with bottom ash and the combined ash is often disposed in landfills [9].

There have been efforts to identify the beneficial utilization of MSWI BA in the construction sector. Cho et al. [10] conducted a comprehensive literature review to see the potential of MSWI ashes as construction material. It has been investigated whether MSWI BA can be utilized as a replacement for fine aggregate in HMA (hot mix asphalt). Thus far, research suggests that this may not be economical because of B.A.’s high porosity/absorption [11,12]. Nevertheless, some studies recommend limiting the use of B.A. to lower than 25% [13]. Utilizing the B.A. in road construction can be beneficial as the reuse of waste materials becomes increasingly important, particularly in construction and highway pavements. This is due to the decrease in resources of virgin aggregates, an increase in transport distances, and a decrease in landfill space [14]. As road materials, Tasneem et al. [15] investigated the leaching behavior of the MSWI-BA-mixed HMA and reported that the toxic level from the leachate is lower than the environmental regulations.

Recycled asphalt shingle (RAS) is another notable source of solid waste materials generated from the construction–demolition debris sector. In the U.S., eleven million tons of RAS are generated annually, accounting for a fraction of the total MSW generated, amounting to around 250 million tons. Post-consumer scrap, represented by tear-off shingles, constitutes 90% of this waste, while post-manufacture scrap represents the remaining 10% [2]. Among the states with the highest shingle production rates, California generates approximately 1.2 million tons of shingles annually, of which 1.1 million tons of tear-off shingles arise from roof replacement projects [3].

The predominant disposal mode of roofing shingle waste Is landfilling, estimated at between USD 18 and USD 60 per ton [4]. Figure 1b illustrates, as an example, the composition of construction and demolition (C&D) materials produced in the state of Florida. Asphalt shingles (tear-off shingles) constitute approximately 7% of the C&D waste on a weight basis. According to previous studies, assuming that the processed shingles have a 20% asphalt composition, the addition of 5% shingles can result in a 1% reduction in the total virgin liquid asphalt content in hot mix asphalt (HMA). Generally, those asphalt shingles comprise fine aggregate, aged asphalt, fibers, etc.

Nam et al. [16] conducted a study on the mechanical properties of shingle-mixed HMA and reported a decrease in rut depth from 3.7 mm to 1.4 mm with the addition of 5% shingle. Moreover, they observed a reduction in the optimal virgin binder content from 5.77% to 4.77% with a 5% shingle addition. Another study has shown that the grounded shingle was used in cement mortar and the fiber in the shingle helps to gain the strength of concrete [17]. Cooper et al. [18] checked the concentration of asphaltene in the extracted aged binder from the shingle. The results indicated that the asphaltene content in the RAS-derived asphaltenes was much greater than that of a regular binder.

Furthermore, the considerable variation in their molecular weight distributions suggested that it is necessary to consider binder compatibility when using RAS-derived binders. Wu et al. [19] performed a rutting test on HMA specimens mixed with RAS. Due to the presence of the aged binder of the shingle, the test results indicated that the RAS-mixed asphalt mixture has a much lower rut depth than the control mixture. Similarly, Cascione et al. [15] evaluated the fatigue behavior based on a four-point bending beam and found that the RAS-mixed specimens showed comparable fatigue performance. Willis et al. [20] proposed that using RAP and RAS in HMA can significantly reduce the construction cost by up to 35%.

Some of the studies have also investigated the positive effect of nanoclay in asphalt mixture [21,22] and potential use of those recycling materials in geo-applications [23,24]. Particularly, ash has been used in stabilizing subgrade soils of pavement systems [25,26].

The previous studies are limited to the use of individual recycling material in HMA. The impact of each material alone was well characterized, but the combined use of both recycling materials was not investigated, which was the research gap. As reported, MSWI BA has high porosity and absorption; thus, the optimum asphalt content is increased when the B.A. is used in HMA. RAS that contains about 30–40% aged binder may compensate for this limitation. Therefore, the novelty of the presenting study is the combined use of two recycling materials (MSWI BA and RAS) so that the mechanical performance of the asphalt mixture is enhanced and the sustainability efficiency is maximized in resource conservation and reduced CO_2_. The main hypothesis of our study is that MSWI BA, due to high porosity, requires a higher amount of asphalt binder in the mixture and that the aged binder in RAS can make up this limitation. The combined use of the MSWI BA and RAS can compensate each other and the performance of the BA-RAS asphalt mixture can be maximized. Therefore, the research objective is to investigate the effect of the combined use of MSWI BA and RAS in HMA, investigating the mechanical performance of the HMA.

## 2. Methodology and Scope

**Figure 2:**
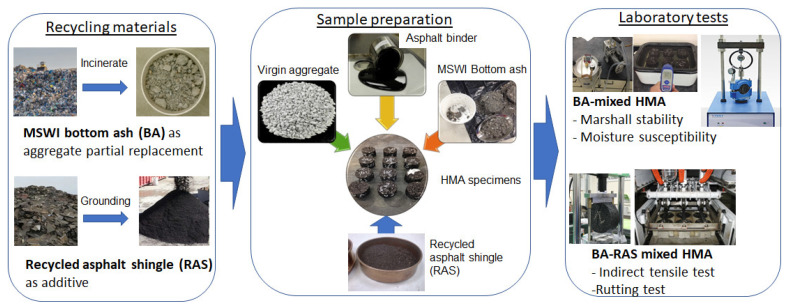
Flowchart of the research program.

In this study, we employed MSWI BA as the aggregate and RAS as the additive in HMA. Figure 2 shows the flow chart of the research methodology. MSWI BA was used to partially replace the virgin aggregate and the ground RAS was used as an additive. Based on the comprehensive literature review, a typical range of the aggregate replacement and admixture content was adopted for the two recycling materials. MSWI BA was used to partially replace the fine aggregate with 10, 20, 30, and 40%. RAS as admixture was added to the mixture with 1, 2, 3, 4, 5, and 6% content. This mixing scenario aimed to identify the optimum proportioning of the MSWI BA and RAS in the asphalt mixture. The HMA mixtures were prepared and then tested by multiple laboratory tests to evaluate their mechanical performance. As seen in Figure 2, the laboratory tests included the Marshall test, indirect tensile test, moisture susceptibility test, and rutting test (asphalt pavement analyzer). Table 1 summarizes all cases of specimen preparation. The sample I.D. represents the material component and percentage of either replacement or addition. For example, the sample code of “V100-B20-A5.7-R2” means that the mixture contains 100% of virgin coarse aggregate (V), 20% of bottom ash (B), 5.7% of asphalt content (A), and 2% of recycled asphalt shingle (R).

## 3. Materials

### 3.1. Asphalt Binder

The study used PG 67–22 asphalt binder, which is commonly used in Florida, U.S., due to its suitability for warm weather conditions. Table 2 summarizes the physical properties of the asphalt, including viscosity, dynamic shear modulus, penetration, and flash point. It is noted that the base binder used in the study was not modified [27,28,29,30,31].

### 3.2. Aggregate and Recycling Materials

Limestone, which is common in Florida (rich limestone bedrock), was selected as the virgin aggregate for the HMA mixture. The maximum size was 25 mm. The limestone aggregate was obtained from a local supplier in Orlando, Florida. The fine aggregate was also made of limestone, obtained by fracturing larger particles.

The MSWI BA used in this study was found to be lightweight, porous, and absorbent with a grayish appearance. The MSWI BA was obtained from a refuse-derived fuel (RDF) incineration facility in Florida. Prior to sieving, manual separation was carried out to remove unburned organics (e.g., glass, metal, paper, etc.). An example photo of the MSWI BA can be seen in Figure 2 (center diagram). The MSWI BA passing sieve No. 4 (smaller than 4.75 mm) was used to replace the virgin fine aggregate. Table 3 summarizes the physical properties of the MSWI BA, including specific gravity, absorption, unit weight, and L.A. abrasion loss [32,33,34]. The references of the standard testing methods are presented in the table.

RAS was also obtained from one of the local manufacturers in Orlando, Florida. The basic physical properties of RAS were tested and visually inspected. The specific gravity is 2.25 and the fineness modulus is 2.25. The RAS sample includes some impurities, such as wood and granules; thus, they were sieved out with sieve No. 8 (2.36 mm opening size) to remove those impurities.

## 4. Specimen Preparation

### 4.1. Control HMA Specimen

#### Optimum Binder Content (OBC)

The OBC was estimated for the control specimen that contains no MSWI BA and RAS. The heat was applied to aggregates and mixed with liquid binder at different binder content. Our previous studies show that the optimum binder content ranges approximately between 4% and 7%; thus, we tried the binder content from 4% to 6.5% with a 0.5% increment (by wt % of the mixture). The specimens were compacted with a Marshall hammer (75 blows on each side) according to ASTM D6927 [35]. For each mix design (or binder content), we prepared three samples in each case, resulting in a total of 18 samples.

The bulk specific gravity, $G_{mb}$, was computed by:

(1) $$G_{mb}=\frac{W_{D}}{W_{SSD}-W_{sub}}$$

where *W_D_* = weight dry in air, *W_sub_* = weight submerged in water, and *W_SSD_* = weight dry in saturated surface.

The void of total mix (*VTM*) was computed by:

(2) $$VTM=\left(1-\frac{G_{mb}}{G_{mm}}\right)100$$

where *G_mb_* = specimen bulk density and *G_mm_* = max theoretical specific gravity.

We then computed the void filled with asphalt (*VFA*) by:

(3) $$VFA=\left[\frac{VMA-VTM}{VMA}\right]100$$

**Figure 3:**
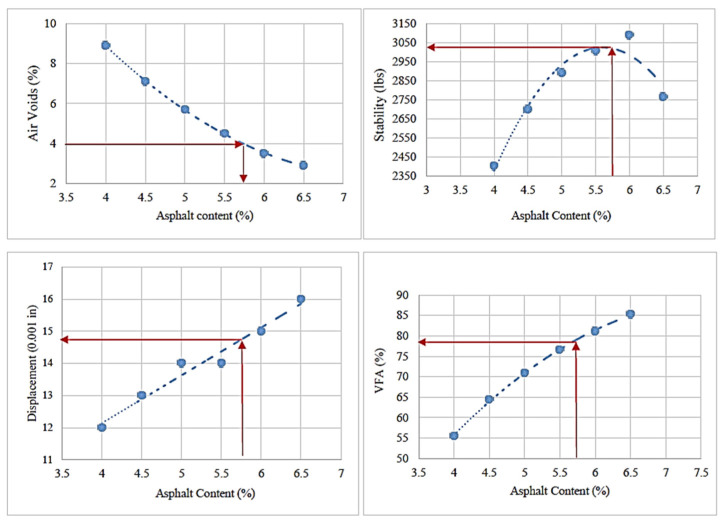
Procedure used to determine the OBC for the control HMA [note: physical/mechanical properties at 4% of air void].

The specimens were then tested to measure stability and flow, followed by ASTM D6927. Figure 3 shows the relationship between binder content and each mixture’s physical/mechanical properties to determine the optimum binder content. Table 4 presents the specification limits for the Marshall mix design. According to the data, the OBC is 5.7% for the control HMA that contains no MSWI BA and RAS.

### 4.2. BA-Mixed HMA

#### 4.2.1. Mix Design

**Figure 4:**
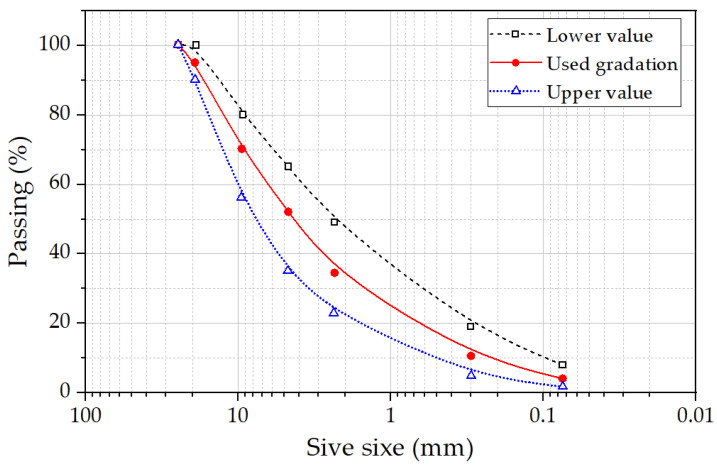
Particle distribution of the aggregate with the limits.

The MSWI BA partially replaced virgin fine aggregate in the asphalt mixture. The bottom ash was sieved out with particle sizes smaller than 4.75 mm (passing sieve No. 4). Figure 4 shows the particle distribution of the BA used in the mixture. The HMA specimens were prepared with MSWI BA replacing 0% through 40% (with 10% increments) of the fine aggregate (by total weight). To satisfy the fine aggregate requirements, both BA and limestone aggregates were separated as individual sieve sizes and combined to the target gradation (see Table 4).

We adopted a smaller specimen size, a 4 in. diameter of HMA, because of the limited amount of MSWI BA. Therefore, the Marshall mix design was used for the sample design. Three specimens were made for each mix design and the average value was reported as the representative value. HMA specimens with 0% MSWI BA, which is referred to as the “control mix”, were made to investigate the optimum binder content (OBC) for the limestone aggregate, which is 5.7% asphalt content. Subsequently, the HMA mixtures with BA 10%, 20%, 30%, and 40% were prepared at the OBC (5.7%). Table 5 is organized for details of the aggregate replacement by the MSWIBA.

#### 4.2.2. Optimum Binder Content (OBC)

**Figure 5:**
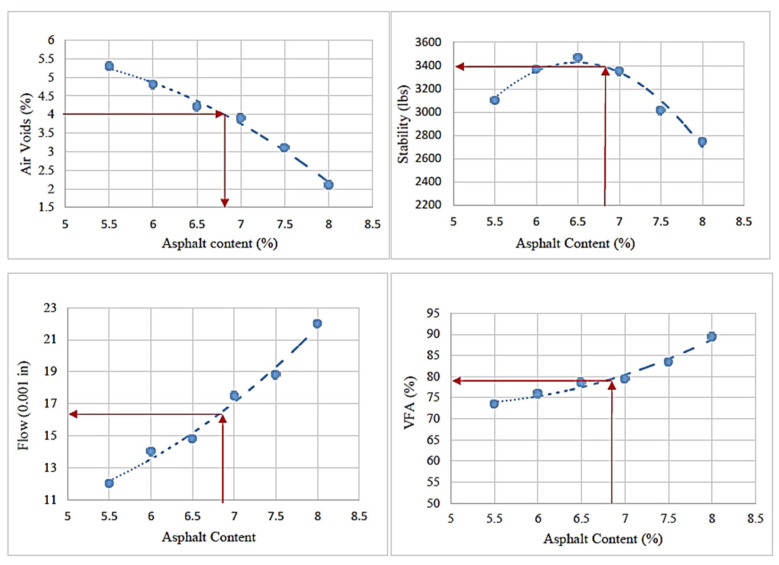
Procedure used to determine the OBC for BA (20%)-mixed HMA [note: physical/mechanical properties at 4% of air void].

Based on the Marshall and moisture susceptibility tests results, it was determined that a 20% aggregate replacement by the BA was optimal for the mixture. MSWI BA is a lightweight aggregate with high absorption and porosity; thus, a greater amount of asphalt binder is necessary to maintain the desired film thickness. Thus, we investigated the optimum binder content for the 20% BA mixture. The Marshall mix design shown in Figure 5 was used. The optimal binder content is determined by evaluating the mixture’s stability, air voids, flow, and void filled with asphalt (VFA). The procedure shown in Figure 5 identified an optimal binder content of 6.8%. The control mix (0% BA) and the 20% BA mix indicate optimal binder contents of 5.7% and 6.8%, respectively. The 20% BA requires additional asphalt binder of 1.1% based on the Marshall method. Even with a 5.7% binder content, all the requirements are met. Table 6 indicates specification of the optimum binder content at 20% MSWI BA replacement.

### 4.3. BA-RAS-Mixed HMA

#### Mixture Design

As the MSWI BA replaces 20% of the virgin fine aggregates, the optimum asphalt content (OAC) was increased by 1.1% (wt%). RAS was added into the HMA mixture at various percentages to mitigate this increase by the additional binder from RAS. At a fixed asphalt content of 5.7%, we added the RAS, ranging from 1% to 6% with an increment of 1%, into the HMA. Table 7 shows the composition of the BA-RAS-mixed HMA specimens.

## 5. Experimental Program

Details of the laboratory testing methods are presented herein. It is noted that the Marshall and moisture susceptibility test characterized the BA-mixed HMA, while the BA-RAS-mixed HMA was tested using indirect tensile strength and rutting tests.

### 5.1. Marshall Test

The Marshall stability and flow test was carried out (ASTM D 6927) [35]. We employed 75 blows by the Marshall hammer to achieve the target compaction level. The values of “flow” and “stability” from the Marshall test represent the displacement and strength characteristics of the mixture, respectively. For each test, three specimens were prepared and the test results were then averaged for the representative value. A loading rate of 50.8 mm/min was employed.

### 5.2. Moisture Susceptibility Test

**Figure 6:**
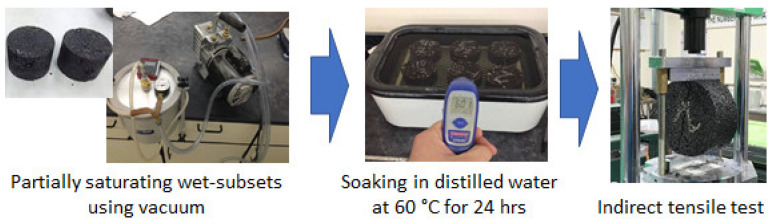
The procedure of the moisture susceptibility test.

The Lottman test was carried out for the moisture susceptibility assessment (ASTM D4867) [36]. This test investigates the negative influence of water on the mixture’s tensile strength. Each set of samples was divided into two groups with a similar air void content (71%) after testing. One group was kept dry while the other was exposed to moisture, with the test being conducted at a temperature of 25 °C. The moisture susceptibility test method, as shown in Figure 6, was utilized to evaluate the impact of water on the tensile strength of HMA paving. Dry samples were sealed and kept in a water bath at 25 °C, whereas wet samples were partially saturated before being soaked in distilled water for 24 h at 60 °C and 1 h at 25 °C to maintain the temperature. The indirect tensile test (IDT) equipment was utilized to determine the indirect tensile strength of each sample at a strain rate of 2 inches per minute. The formula for calculating the indirect tensile strength was as follows:

(4) $$S_{t}=\frac{2P}{\pi tD}\;\left[psi\right]$$

where *P* = maximum loading, *S_t_* = tensile strength, *t* = thickness of specimen, and *D* = diameter of specimen.

(5) $$TSR=\left(\frac{S_{tm}}{S_{td}}\right)100\;\left[\%\right]$$

where *TSR* = tensile strength ratio, *S_tm_* = average tensile strength of the moisture sample, and *S_td_* = average tensile strength of the dry sample.

Many highway agencies (e.g., state DOTs) normally require the use of anti-strip agents for asphalt pavements. FDOT’s regulations specify that an antistrip agent can be blended with an asphalt binder at a concentration ranging from 0.25% to 0.50% by weight. In this study, an anti-strip agent was not used in order to better investigate the sole effect of the BA content on moisture-induced damage.

### 5.3. Indirect Tensile Strength (IDT) Test

The cylindrical specimen was loaded diametrically, as seen in Figure 2. For the loading, a strain rate of 2 in/min. was employed. The perpendicular deformation of the loading direction results in tensile failure. The maximum load and specimen dimensions were recorded to calculate the material’s indirect tensile strength using Equation (4).

### 5.4. Rutting Test (Asphalt Pavement Analyzer Test)

The rutting resistance of each specimen was investigated and the asphalt pavement analyzer (APA) test was employed. This test was chosen to ensure the comparability of the data since the APA measurement assesses mixture stiffness and measures sample rut depth directly. The testing was performed on 75 mm dry compacted HMA cylindrical samples that have 7.0 ± 0.5 percent air voids. The specimens were tested under 64 °C and 8000 cycles of loading were applied. Rutting is a complex phenomenon influenced by various factors, according to the literature on the subject.

## 6. Results and Discussion

### 6.1. BA Mixed HMA

#### 6.1.1. Marshall Test

**Figure 7:**
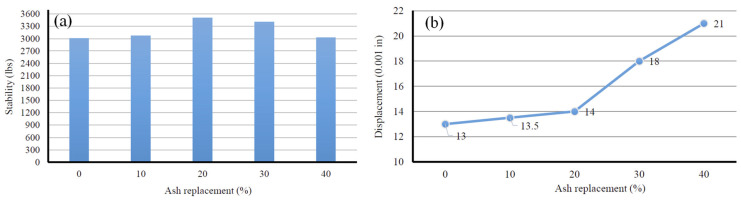
Marshall testing result for the MSWI-BA-mixed HMA: (**a**) stability and (**b**) flow.

The results of Marshall testing are shown in Figure 7. The stability was observed to increase by 2%, 16.5%, 13.3%, and 0.5% with the addition of BA in the mixture at 0%, 10%, 20%, and 40% by weight, respectively. Similarly, the flow was observed to increase by 4%, 8%, 38%, and 61% as the amount of bottom ash in the mixture increased from 0% to 10%, 20%, and 40%, respectively. The increase in stability of the bottom ash aggregate was attributed to the surface roughness of the particles, which enhanced the interlocking between the particles. However, beyond the 20% replacement, the stability began to decrease due to a decrease in the binder content.

#### 6.1.2. Moisture Susceptibility Test

**Figure 8:**
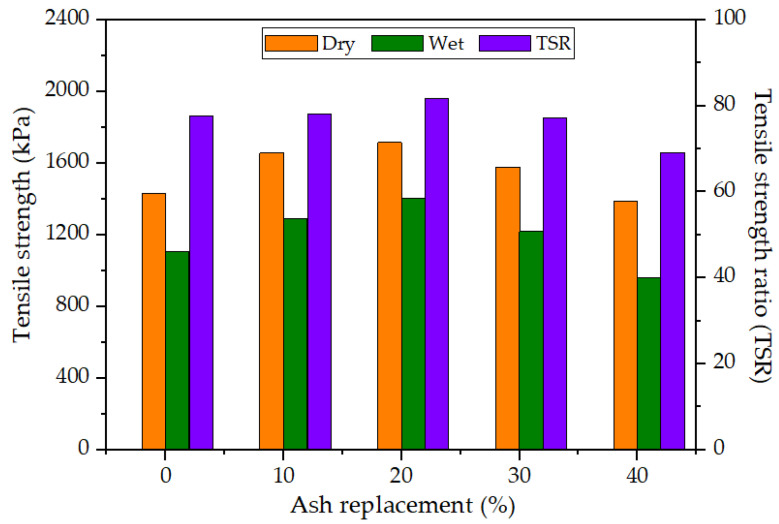
Tensile strength test results: dry versus wet samples, tensile strength ratio (TSR).

Figure 8 depicts the findings of the moisture susceptibility test, wherein it can be observed that the 20% replacement of MSWI BA displays the greatest tensile strength of 1720 kPa, indicating an increase of 288 kPa compared to the control specimen with a tensile strength of 1432 kPa. All MSWI-BA-combined HMA samples exhibit a tensile strength greater than the control, except for the 40% MSWI BA substitution. However, only the HMA mixtures with a 10% and 20% BA substitution exhibit greater tensile strength ratios (TSRs) than the control specimen, as per the results of the test, indicating that they are better suited for use in practical applications. Most highway agencies require the minimum criterion of 80%; thus, based on the criterion, only the 20% replacement of MSWI BA satisfies the requirement. This is attributed to the fact that MSWI BA is more porous compared to conventional aggregates such as limestone and granite, leading to increased water absorption. Consequently, the utilization of MSWI BA in the HMA mixtures may result in a reduced effective asphalt binder content, thereby diminishing the HMA’s ability to withstand moisture. Notably, the study did not assess the cracking resistance of the HMA mixtures, and thus it is plausible that the mixtures may exhibit low cracking resistance despite the observed enhancements in stability and TSR up to a 20% MSWI BA substitution.

### 6.2. BA-RAS-Mixed HMA

#### 6.2.1. Indirect Tensile Strength Test

**Figure 9:**
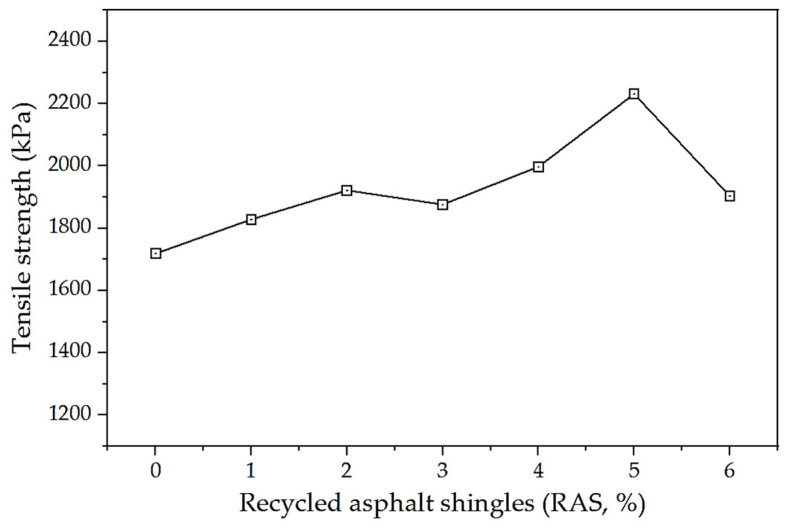
Results of the tensile strength test (BA-RAS-mixed HMA specimens at fixed 20% of BA).

The 20% MSWI BA replacement resulted in an increase of 1.1% (wt%) of the optimum asphalt content. In order to minimize this percentage increase, RAS was incorporated into the trial mixture in varying amounts of 1 through 6 percentage points based on the total aggregate mass. The outcomes of this experiment are presented in Figure 9.

Incorporating RAS into the HMA blend enhances the binder’s tensile strength, with a 29.5% higher increment observed at a 5% ratio compared to a 0% ratio. However, the excessive addition of binder causes the mixture to become more ductile, resulting in a 6% reduction. Moreover, a 6% inclusion of RAS has the potential to replace the quantity of fine aggregates in the mixture by up to 60%. As a consequence, the mixture transforms from a viscoelastic state to a plastic state with the incorporation of additional filler.

#### 6.2.2. Rutting Test

**Figure 10:**
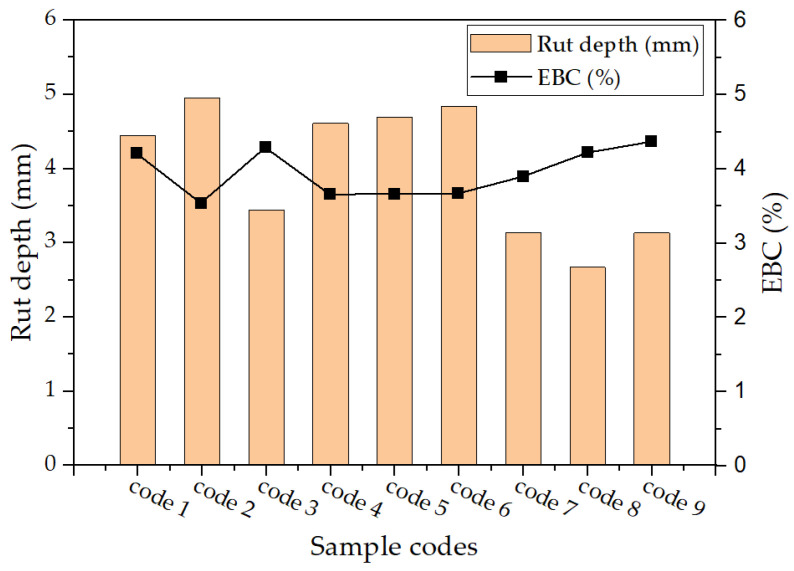
Rutting test result (rut depth vs. effective binder content) (note: code number from Table 1).

The results of the rutting test, as presented in Figure 10, along with the effective binder content (EBC) of the suggested samples, exhibit an irregular pattern inconsistent with the trend observed in the tensile strength outcomes. The variability in the EBC is responsible for this phenomenon, given that the EBC is directly related to the amount of mixture binder. The substitution of 20% of the fine aggregates with MSWI BA reduces the EBC, resulting in lower resistance to permanent deformation, as demonstrated in the specimens labeled “V100-B20-A5.7-R0”. The presence of porosity on the surface of MSWI BA particles means that the addition of 1, 2, and 3 percent of RAS does not have a significant effect on the EBC. Instead, the extra filler materials provided by RAS enable greater plastic deformation. Samples containing 4, 5, and 6 percent of RAS show an increase in the EBC, which has a greater impact on rut depth than the filler materials in RAS. However, the sample with 6 percent of RAS has only a slightly higher EBC percentage than the control sample, leading to an increase in rutting.

### 6.3. Correlation Analysis between Stiffness and Rut Depth

**Figure 11:**
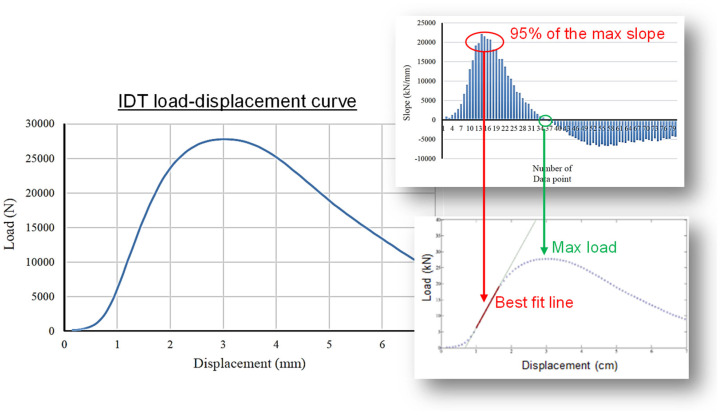
Methodology used to determine the IDT stiffness (k value).

A new stiffness index has been introduced to establish the correlation between stiffness and rut depth. The stiffness is computed by identifying the slope of the load–displacement curve, as seen in Figure 11. This slope is referred to as “IDT stiffness (*k*)”. Steeper slopes (higher *k* values) indicate stiffer mixtures that can be associated with greater resistance to permanent (or plastic) deformation. In order to determine the IDT stiffness (k), an algorithm was developed based on MATLAB (R2020b) software. A tangential line over a small segment was computed and continuously plotted along the entire load–displacement curve. The maximum slope represents the point with maximum stiffness, and the failure point is indicated by a zero slope after the maximum. The mixture displays linear behavior over a section before and after the maximum stiffness under monotonic loading. The data points in the section of 95% of the maximum slope are chosen for Polyfit, and the best-fit line is obtained (see Figure 11). This best-fit line is then denoted as the “k” value.

**Figure 12:**
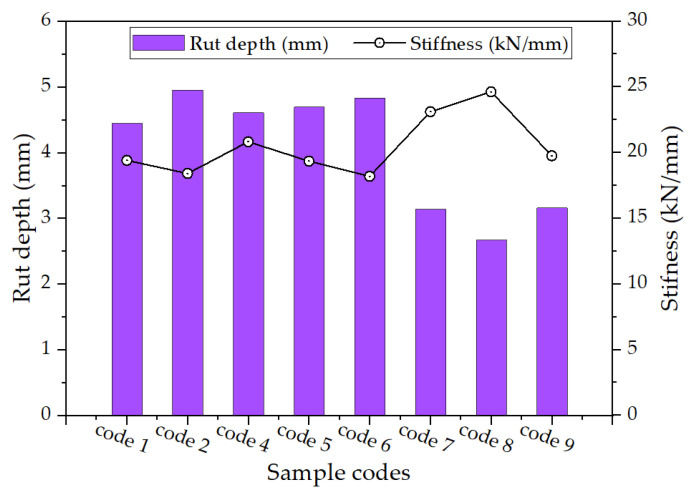
Comparison between the rut depth and stiffness (k = IDT stiffness) (note: code number from Table 1).

The correlation between rut depth and IDT stiffness (k) is illustrated in Figure 12. A higher “k” value corresponds to a smaller rut depth. It is widely known that stiffer materials are more susceptible to fatigue cracking. Thus, the rut resistance and fatigue crack resistance are trade-offs (see Figure 12).

## 7. Conclusions

Unlike the previous studies that focused on the individual use of recycling material in HMA, the presented study explored the beneficial use of MSWI BA and RAS in HMA, particularly their combined effect on mechanical performance. RAS was used as an additive and the MSWI BA was used as a replacement for aggregate in HMA. Our main hypothesis is that the increased optimum asphalt binder, due to the high porosity of MSWI BA, will be supplemented by the extra asphalt binder from RAS. It is important to note that MSWI BA’s high absorption will require a higher amount of asphalt binder in order to provide sufficient asphalt film (or coating) around the aggregate when compared with normal aggregates such as limestone, granite, etc. However, RAS can supply those required asphalt binders because it has an about 30–40% binder component. The following conclusions have been drawn based on a series of laboratory tests.

- The control HMA (0% MSWI BA, 0% RAS) indicates the optimum binder content as 5.7%.
- The optimum replacement of the fine aggregate is 20% MSWI BA in HMA and the 20% BA-mixed HMA shows its optimum binder content as 6.8%. Thus, the 20% replacement of the MSWI BA increases the optimum binder content of the mixture by 1.1%.
- When the 20% MSWI BA replacement is fixed, as the RAS content increases, the strength of the HMA increases. A 5% addition of RAS exhibits the greatest strength of the mixture.
- All mixtures demonstrated greater strength than the control specimen (regular HMA), indicating a greater mechanical performance than typical HMA.

Based on the findings, it is believed that the combined use of MSWI BA and RAS in HMA is a cost-effective alternative and that their mechanical performance is compatible with normal HMA mixtures. Therefore, the combined use of MSWI BA and RAS in HMA is preferred over individual usage. It was also observed that the asphalt mixture exhibits the best mechanical performance at a 20% aggregate replacement by MSWI BA and 5% RAS as an admixture.

As a future study, one may quantitatively evaluate not only cost–benefit analysis (including life cycle cost analysis) but also life cycle assessment (LCA) for the combined use of MSWI BA and RAS in HMA prior to their field implementation. Additionally, the authors understand that the current study was limited to basic mechanical performance such as strength and stiffness; therefore, future studies may evaluate fatigue and fracture performance as part of ongoing research.

## Figures and Tables

**Table 1:** Mixture specimens and the sample I.D. (Note: OBC = optimum binder content, AC = asphalt content).

Code Number	Sample ID	Description
Code 1	V100-B0-A5.7-R0	100% virgin coarse and fine aggregates @ 5.7% OAC
Code 2	V100-B20-A5.7-R0	100% virgin coarse + 80% virgin fine+20% BA fine agg. @ 5.7% AC
Code 3	V100-B20-A6.8-R0	100% virgin coarse + 80% virgin fine+20% BA fine agg. @ 6.8% AC
Code 4	V100-B20-A5.7-R1	100% virgin coarse + 80% virgin fine+20% BA fine agg. + 1% RAS @ 5.7% AC
Code 5	V100-B20-A5.7-R2	100% virgin coarse + 80% virgin fine+20% BA fine agg. + 2% RAS @ 5.7% AC
Code 6	V100-B20-A5.7-R3	100% virgin coarse + 80% virgin fine+20% BA fine agg. + 3% RAS @ 5.7% AC
Code 7	V100-B20-A5.7-R4	100% virgin coarse + 80% virgin fine+20% BA fine agg. + 4% RAS @ 5.7% AC
Code 8	V100-B20-A5.7-R5	100% virgin coarse + 80% virgin fine+20% BA fine agg. + 5% RAS @ 5.7% AC
Code 9	V100-B20-A5.7-R6	100% virgin coarse + 80% virgin fine+20% BA fine agg. + 6% RAS @ 5.7% AC

**Table 2:** Properties of the asphalt binder.

Test	Method	Specification	Results
Rotational viscocity @ 135 °C, 20rpm spindle #21	AASHTO T316 [27]	3.0 Max	0.456 Pa.s
Rotational viscocity @ 165 °C, 20rpm spindle #21	AASHTO T316 [27]	3.0Max	0.128 Pa.s
Dynamic shear (G*/sinδ, 10 rad/s)	AASHTO T315 [28]	1.0 min @ 67°	1.09 kPa
Ring and ball soft point	AASHTO T53 [29]	-	54 °C
Penetration @ 25 °C	AASHTO T49 [30]	-	59 dmm
Flash point	AASHTO T48 [31]	230 °C	344 °C

Note: Pa.s = pascal-second.

**Table 3:** Properties of MSWI bottom ashes and limestone aggregate.

Properties	Limestone *	MSWI Bottom Ash
Specific gravity (oven dry) ASTM C127 [32]	2.4	2.2
Absorption capacity ASTM C127 [32]	3.04%	12.8%
Unit weight (oven dry) ASTM C29 [33]	n/a	2195 kg/m^3^
L.A. abrasion mass loss ASTM C53 [34]	36%	43%

* Limestone as a reference material.

**Table 4:** Optimum asphalt binder specification limits.

Test Property	Specification	Results
Marshall stability (1 bf)	1500 (minimum)	3005
Flow 0.01 inch	8–16	14.8
Void in total mix (%)	3–5	4
Void filled with Asphalt cement	70–80	78

**Table 5:** Details of the aggregate replacement by the MSWI BA (note: the value of each cell represents the weight of each fraction).

Sieve No. (Size)	Replacement Ratio of BA				
	0%	10%	20%	30%	40%
	Virgin Agg (g)	BA (g)	BA (g)	BA (g)	BA (g)
19 mm	58	0	0	0	0
12.5 mm	173	0	0	0	0
9.5 mm	115	0	0	0	0
#4	207	0	0	0	0
#8	201	20	40	60	80
#16	109	11	22	33	44
#30	92	9	18	28	37
#50	75	7	15	22	30
#100	40	4	8	12	16
#200	31	3	6	9	12
Pan	49	5	10	15	20

**Table 6:** Specification of the optimum binder content at 20% MSWI BA replacement (note: same specifications in Table 4 but different testing results).

Test Property	Specification	Results
Marshall stability (1 bf)	1500 (minimum)	3080
Flow 0.01 inch	8–16	16.2
Void in total mix (%)	3–5	4
Void filled with Asphalt cement	70–80	78

**Table 7:** Composition of the BA-RAS-mixed HMA.

Asphalt Content	Coarse Aggregate	Fine Aggregate	RAS as Admixture
5.7%	limestone aggregate	20% replacement by MSWI BA	1, 2, 3, 4, 5, 6%

## Data Availability

Data are contained within the article.
